# Gubernaculum Sparing Laparoscopic Orchiopexy in Cryptorchidism with Ipsilateral Congenital Absence of the Vas Deferens: Unique Outcome

**DOI:** 10.1155/2019/7408412

**Published:** 2019-03-25

**Authors:** Ebtehal Althobaiti, Hattan Badr, Maryam Aloqalaa, Raghdah Alsharif, Naif Alqarni

**Affiliations:** ^1^Faculty of Medicine, Umm Al-Qura University, Makkah, Saudi Arabia; ^2^Department of Urology, King Abdullah Medical City, Makkah, Saudi Arabia; ^3^Pediatric Urology Division, Pediatric Surgery Department, Maternity and Children Hospital, Makkah, Saudi Arabia

## Abstract

Congenital absence of the vas deferens (CAVD) is an uncommon anomaly that occurs in up to 1% of the male population. It can be associated with various other anomalies, including cryptorchidism and renal anomalies, such as renal agenesis. We here present a case of cryptorchidism with ipsilateral congenital absence of the vas deferens and renal agenesis and used the Stephen-Fowler technique for Orchiopexy depending on gubernacular vessels. A 7-month-old boy was referred to our center with left grade 2 hydronephrosis, right renal agenesis, and right impalpable, undescended testis. Examination under anesthesia and laparoscopic exploration with staged Stephen-Fowler orchiopexy were performed. The patient was followed up at 3, 6, and 12 months and had an excellent outcome. Cryptorchidism with congenital ipsilateral absence of the vas deferens and renal agenesis is a rare diagnostic entity. Our case supports the suggested theory that the gubernacular vessels can increase the blood supply to the testis, although further studies are needed to confirm this hypothesis.

## 1. Introduction

Congenital absence of the vas deferens (CAVD) is an uncommon anomaly that occurs in only about 1% of the male population [1]. Since the development of the genital and renal systems are closely integrated, renal agenesis and other renal anomalies often coexist with CAVD, in about 30.2% of cases [2]. Although the exact spectrum of causes is unknown, it involves a defect in the developmental process before the complete separation of the Wolffian duct (mesonephric duct) and ureteric bud during the 5th week of gestation [3]. Such anomalies are usually found incidentally in the evaluation of various conditions, including undescended testis, which is considered one of the most common congenital anomalies in boys [4].

In 1976, a laparoscopic procedure was initiated for the diagnosis of impalpable, undescended testis; this has now become the gold standard as a diagnostic and therapeutic procedure [4]. One of the most commonly used laparoscopic procedures for high intra-abdominal testes is the staged Stephen-Fowler orchiopexy which has had a success rate reaching up to 100%, as reported by Agrawal et al. [5]

We here present a case of cryptorchidism with ipsilateral CAVD and renal agenesis and used the Stephen-Fowler technique for Orchiopexy depending on the gubernacular vessels, with an excellent outcome.

## 2. Case Presentation

A 7-month-old boy was referred to the Maternity and Children Hospital in Makkah for further management of left grade 2 hydronephrosis, right renal agenesis, and right impalpable, undescended testis. The patient presented to the outpatient department (OPD) for assessment. Genital examination revealed a normal-size, circumcised penis, sizable left testis in the scrotum, and an impalpable, undescended right testis. The patient was booked for examination under anesthesia and laparoscopic exploration.

**Figure 1:**
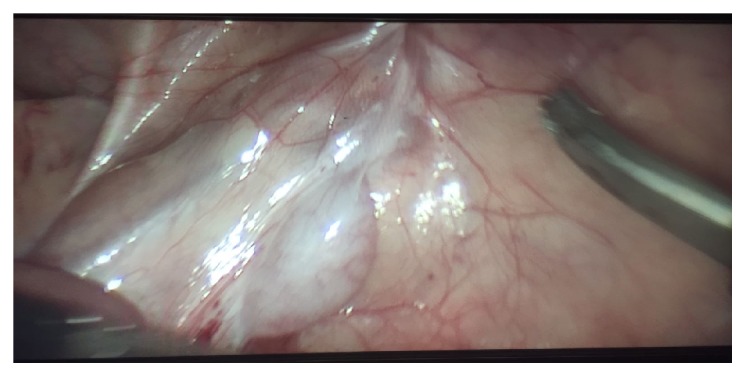
Absence of the right vas deferens in laparoscopic surgery.

Laparoscopic exploration was performed on February 13, 2017, and revealed a sizable right testis proximal to the internal inguinal ring by 4 cm with short spermatic vessels, and an absent right vas deferens and epididymis (Figure 1). This left the choice of performing either Orchiectomy or Orchiopexy. For the sake of hormone generation, we decided to perform Orchiopexy depending on the gubernacular vessels. We therefore performed first-stage Stephen-Fowler Orchiopexy by clipping the spermatic vessels.

**Figure 2:**
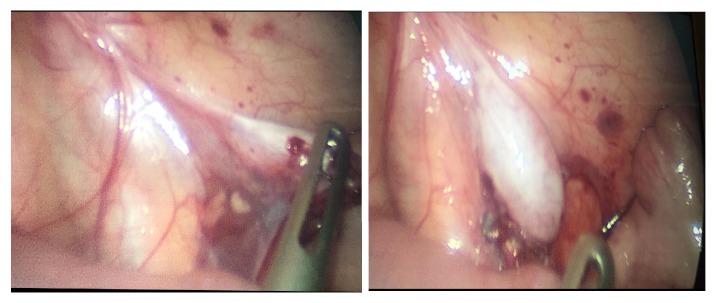
Thicker gubernacular vessels in the 2^nd^ stage Stephen-Fowler compared to the 1^st^ stage.

**Figure 3:**
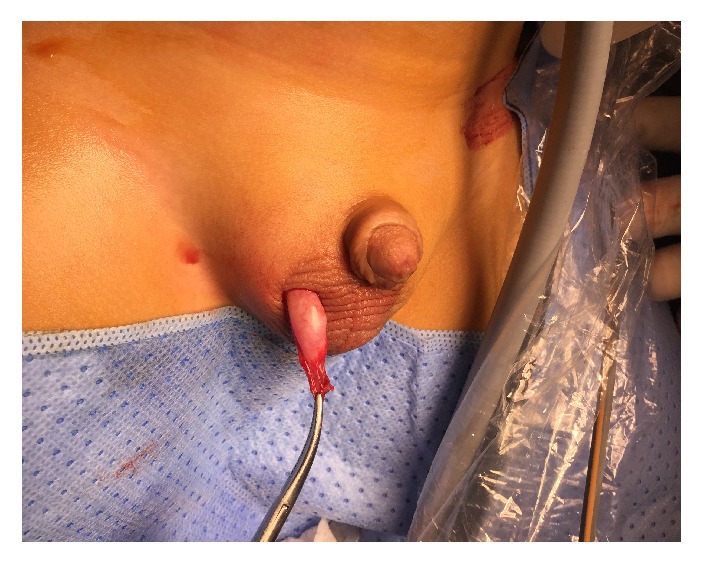
Fixation of the right testis into the scrotum.

After 6 months, the patient was admitted for second-stage Stephen-Fowler orchiopexy on September 11, 2017. Intraoperative findings showed that the testis was the same size. We performed peritoneal dissection lateral to the testis; the medial peritoneal aspect and the gubernaculum remained untouched (Figure 2). The testis was brought through the inguinal canal into the right hemiscrotum, where a dartos pouch was created (Figure 3) and the testis was fixed in this position. The dimension of the testis was 12 × 5 mm.

**Figure 4:**
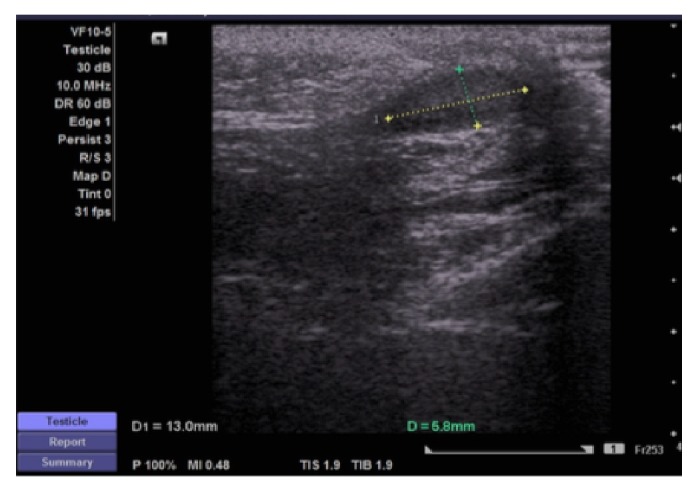
US of the right testis at six-months follow-up.

At three-month follow-up, the right testis was assessed in the OPD; both testes were present in the scrotum with normal sensation, and the right testis was comparable in size to the left. Six months after the 2^nd^ stage procedure, ultrasound of the scrotum was performed to assess the size, echogenicity, and vascularity of the testis; this revealed a normal anatomical location of both testicles, with a homogenous echo pattern and normal vascularity. The right testis measured 13 × 6 mm and the left testis measured 16 × 8 mm (Figure 4).

**Figure 5:**
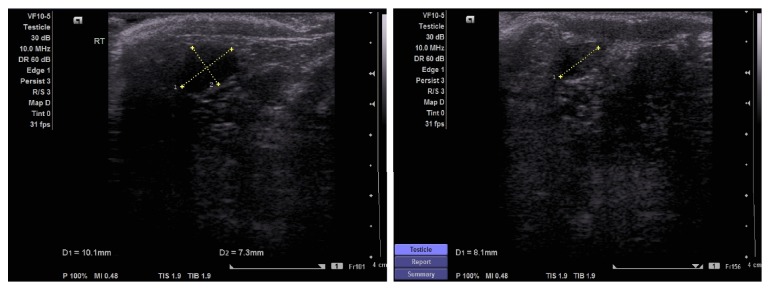
US of the right testis at twelve-months follow-up.

At the 12-month follow-up, both testicles appeared to have a normal anatomical location and parenchymal echogenicity. The right testicle measured 10 × 7 × 8 mm, and the left testicle measured 15 × 8 × 10 mm (Figure 5). Vascularity was good according to doppler ultrasound.

## 3. Discussion

The renal and genital systems codevelop. During the 5^th^ week of gestation, the mesonephric duct gives rise to the ureteric bud, which forms the metanephric duct that later forms the ureter and the metanephric blastema, which produces the standard lobulated feature of the kidney [6]. The mesonephric duct regresses in females but persists in males to outline the genital duct system, which consists of the seminal vesicle, the ductus deferens, and the distal two-thirds of the epididymis [7]. Any interference before the complete division of the mesonephric duct and the ureteric bud during the 7^th^ week or earlier can result in a congenital unilateral absence of the vas deferens (CUAVD) and renal agenesis, as in our case [2, 3].

In 1737, John Hunter first described CUAVD, and Reverdin described the association between agenesis of the vas deferens and the ipsilateral kidney in 1870 [3]. This association is found in 73.3% of cases of CUAVD [2]. Undescended testis has an incidence of up to 3% [4]. The combination of CUAVD and renal agenesis in cryptorchidism in a single case, such as ours, is considered a very rare entity [8].

The Stephen-Fowler procedure reflects a forward extension of the diagnostic laparoscopy and is considered the gold standard, as it is safe, achievable, effective and has a good outcome overall [4]. The procedure is divided into single-stage or two-stage procedures, depending on the location of the undescended testis. Our patient underwent a two-stage Stephen-Fowler orchiopexy, which is used when the testis is close to the iliac vessels or 2 cm away from the internal inguinal ring [9]. In the first stage, the testicular vessels are isolated and clipped at the furthest point from the testis; then the second stage takes place after at least 4 months, in which the peritoneum is dissected around the vessels up to the testis, keeping the testis attached only to the vas deferens which eases the mobilization of the testis as far as the length of the vas will allow [9]. Since our patient had cryptorchidism with ipsilateral absence of the vas deferens, we could not depend on the collaterals from the vasal artery, as would normally be the case. A similar case report was published by Dong et al. who performed laparoscopic orchiectomy due to the short length of the spermatic cord structure, unlike our case, who underwent laparoscopic orchiopexy with dependence on the collaterals from the gubernacular vessel [6]. Lane et al. reported the case of an 18-month-old boy with left renal agenesis, left cryptorchidism, and left absence of the vas deferens; a 6-month follow-up after Orchiopexy showed that the testis reascended into the groin and left Orchiectomy was then performed [7]. Kulkarni et al. reported a 12-year-old boy with right renal agenesis, right cryptorchidism, and right absence of the vas deferens, for whom open Orchiopexy was performed; no follow-up results were described [8].

A study published in 2007 reported an outcome of laparoscopic two-stage Stephen-Fowler Orchiopexy preserving the gubernacular vessels in the presence of vas deferens and stated that the gubernaculum is thick and well vascularized and thus could theoretically increase the blood supply to the testis. They recorded a testicular survival rate close to 90% [10]. We applied this theory to our case and achieved an excellent outcome over the follow-up period.

A recent study first compared gubernaculum sparing laparoscopic orchiopexy (GSLO) and the conventional laparoscopic orchiopexy (CLO). This study prospectively reviewed 212 intra-abdominal testes managed in either way. They reported an overall rate of testicular atrophy of 6.6%, 0.6% in the GSLO group, and 28.3% in the CLO group [11].

## 4. Conclusion

CAVD is a rare anomaly that is associated with other genitourinary malformations. In our case, we depended on the gubernaculum vessels to at least offer the chance for Orchiopexy rather than proceed directly to Orchiectomy. The outcome of our patient supports the theory of Robertson et al. that the gubernacular vessels are able to increase the blood supply to the testis. Further cases should be studied to confirm this hypothesis.
